# PANTHER: AZD8931, inhibitor of EGFR, ERBB2 and ERBB3 signalling, combined with FOLFIRI: a Phase I/II study to determine the importance of schedule and activity in colorectal cancer

**DOI:** 10.1038/s41416-022-02015-x

**Published:** 2022-11-09

**Authors:** David J. Propper, Fangfei Gao, Mark P. Saunders, Debashis Sarker, John A. Hartley, Victoria J. Spanswick, Helen L. Lowe, Louise D. Hackett, Tony T. Ng, Paul R. Barber, Gregory E. Weitsman, Sarah Pearce, Laura White, Andre Lopes, Sharon Forsyth, Daniel Hochhauser

**Affiliations:** 1 Barts Cancer Institute, Queen Mary, University of London, John Vane Science Centre, Charterhouse Square, London, EC1M 6BQ UK; 2 UCL Cancer Institute, Paul O’Gorman Building, University College London, London, WC1E 6DD UK; 3 The Christie, Manchester, M20 4BX UK; 4 School of Cancer and Pharmaceutical Sciences, King’s College London, London, WC2R 2LS UK; 5 UCL ECMC GCLP Facility, UCL Cancer Institute, Paul O’Gorman Building, University College London, London, WC1E 6DD UK; 6 Breast Cancer Now Research Unit, Department of Research Oncology, Guy’s Hospital, King’s College London, London, SE1 9RT UK; 7 Richard Dimbleby Laboratory of Cancer Research, School of Cancer & Pharmaceutical Sciences, King’s College London, London, SE1 1UL UK; 8 Cancer Research UK & UCL Cancer Trials Centre, University College London, London, W1T 4TJ UK

**Keywords:** Colorectal cancer, Colorectal cancer

## Abstract

**Background:**

Epidermal growth factor receptor (EGFR) is a therapeutic target to which HER2/HER3 activation may contribute resistance. This Phase I/II study examined the toxicity and efficacy of high-dose pulsed AZD8931, an EGFR/HER2/HER3 inhibitor, combined with chemotherapy, in metastatic colorectal cancer (CRC).

**Methods:**

Treatment-naive patients received 4-day pulses of AZD8931 with irinotecan/5-FU (FOLFIRI) in a Phase I/II single-arm trial. Primary endpoint for Phase I was dose limiting toxicity (DLT); for Phase II best overall response. Samples were analysed for pharmacokinetics, EGFR dimers in circulating exosomes and Comet assay quantitating DNA damage.

**Results:**

Eighteen patients received FOLFIRI and AZD8931. At 160 mg bd, 1 patient experienced G3 DLT; 160 mg bd was used for cohort expansion. No grade 5 adverse events (AE) reported. Seven (39%) and 1 (6%) patients experienced grade 3 and grade 4 AEs, respectively. Of 12 patients receiving 160 mg bd, best overall response rate was 25%, median PFS and OS were 8.7 and 21.2 months, respectively. A reduction in circulating HER2/3 dimer in the two responding patients after 12 weeks treatment was observed.

**Conclusions:**

The combination of pulsed high-dose AZD8931 with FOLFIRI has acceptable toxicity. Further studies of TKI sequencing may establish a role for pulsed use of such agents rather than continuous exposure.

**Trial registration number:**

ClinicalTrials.gov number: NCT01862003.

## Background

Monoclonal antibodies targeting EGFR, either when combined with conventional cytotoxic chemotherapy, or as single agents, produce significant survival prolongation in patients with RAS and BRAF wild type metastatic colorectal cancer (mCRC) [1]. In contrast, small molecules targeting the EGFR pathway, such as gefitinib, have minimal activity as either monotherapy or in combination with chemotherapy [2].

A factor limiting the efficacy of agents targeting the EGFR pathway is the compensatory upregulation of HER2 (Erb-B2) and HER3 (Erb-B3), which can activate bypass pathways and hence mediate resistance. It has been suggested primary or acquired resistance to antibodies targeting EGFR could either be due to HER2 gene amplification or heregulin up-regulation (a HER3 ligand), leading to persistent extracellular signal-regulated kinase signalling and resistance to cetuximab [3, 4]. Preclinical and clinical data have demonstrated HER3 as an escape pathway to EGFR blockade through a compensatory shift to HER3 signalling through the PI3K/AKT pathway [5–7]. AZD8931 is a novel tyrosine kinase inhibitor with equipotent inhibition against EGFR, HER2, and HER3 signalling. AZD8931 provides the opportunity to investigate whether simultaneous inhibition of three ERBB receptor pathways could be of benefit in CRC. In the multicentre randomised Phase II/III FOCUS4-D trial, 32 patients with metastatic colorectal cancers WT for BRAF, PIK3Ca, KRAS and NRAS after first line induction therapy were randomised 1:1 to either AZD8931 40 mg bd continuous dosing regimen vs placebo. There was no PFS benefit of AZD8931 compared to placebo [8]. In a Phase I dose escalation study combining AZD8931 with oxaliplatin and capecitabine chemotherapy in oesophagogastric cancer (DEBIOC), AZD8931 dosing of 20 mg bd 4 days on/3 days off demonstrated an acceptable safety profile in patients.

Both oxaliplatin and irinotecan have shown synergistic activity with AZD8931 in a variety of preclinical models (AstraZeneca, unpublished data). In this trial AZD8931 was assessed in combination with FOLFIRI (irinotecan/5-FU) chemotherapy. This was based on results of Phase III studies in CRC, indicating that cetuximab confers a survival advantage with this regimen [9, 10]. In contrast, two randomised studies using oxaliplatin based chemotherapy with EGFR inhibition (COIN and NORDIC) showed no advantage [9, 11]. There is evidence that in some indications the combination of oxaliplatin with cetuximab could be deleterious with regard to overall survival (OS) [12]. The reasons for this are unclear but may include effects of EGFR inhibition on free radical formation that mediates the cytotoxicity of platinum-based chemotherapy [13].

There is extensive evidence that antibodies targeting EGFR have activity only in tumours expressing wild-type RAS [14–16]. Preclinical data suggest that AZD8931 may have activity in KRAS mutant backgrounds but this is as yet unclear. It was therefore appropriate in this trial to enrol patients with RAS wild-type tumours.

An important issue in determining the efficacy of EGFR inhibition in CRC has been the schedule used. Continuous treatment with TKIs such as gefitinib and erlotinib can result in G1 arrest, which may reduce the effectiveness of agents that are S phase specific such as irinotecan [17]. In contrast, use of high doses of these agents for shorter duration (pulse treatment) inhibit critical downstream signalling pathways without inducing cell cycle arrest [18, 19]. In this study, therefore, we sought evidence of efficacy for pulsed high dose AZD8931 in combination with FOLFIRI in chemotherapy-naive patients with CRC. In addition, we assessed effects of increasing doses of AZD8931 on DNA damage by the single-cell gel electrophoresis (Comet) assay.

The expression level of HER family members is unreliable as a predictive marker for targeted therapies in cancer [5, 20, 21]. HER receptors are able to form alternative dimers and can therefore compensate the loss of function of one receptor during targeted therapies [22, 23]. The HER dimerisation status may therefore be more important than HER receptor expression in determining sensitivity or resistance to therapy [24, 25]. Therefore, the ability to assess the dimerisation receptor pairs within tumours could be useful as a prognostic or predictive biomarker for targeted therapies in metastatic colorectal cancer.

Förster resonance energy transfer (FRET) assays using fluorescence lifetime imaging microscopy (FLIM) have been developed to analyse the interaction between pairs of molecules [26]. This is a gold standard technique for measuring protein proximity within <10 nm range [27]. Fluorescence lifetime imaging (FLIM) is well suited to the analysis of interaction within HER family receptors in cells and FFPE tissues. Using FLIM, it is possible to measure FRET to quantify interactions between HER receptors at nanometre scale to establish the potential role that crosstalk might play in metastatic colorectal cancer. Such data could help characterise or predict response to anti-HER therapy.

Previous work in our group has shown HER2:HER3 crosstalk was required for endogenous feedback HER3 phosphorylation upon cetuximab treatment in metastatic colorectal cancer cells, and co-treatment with cetuximab and lapatinib can limit this effect [28]. In patients with breast cancer, extent of HER2:HER3 dimer formation in tumour blocks predicted likelihood of metastatic relapse after surgery independently of HER2 expression [24]. Furthermore, subset analysis from the COIN trial [9] showed that in patients treated with chemotherapy plus cetuximab, those with increased HER2:HER3 tumour FRET efficiency had superior PFS [29]. Hence, changes in HER2-3 dimerisation were assessed in this trial using an innovative fluorescence resonance energy transfer (FRET) assay.

This trial examined the toxicity and efficacy profile of pulsed AZD8931 in combination with chemotherapy in first-line treatment of RAS WT metastatic colorectal cancer patients and explored potential biomarkers that may predict response to therapy.

## Methods

### Participants and study design

Eligible patients with histologically confirmed non-resectable metastatic RAS wild-type metastatic colorectal cancer, and WHO performance status 0–1 were recruited onto this study. Patients were chemotherapy naive for metastatic disease and had RECIST measurable tumours. Prior adjuvant chemotherapy was allowed provided it was completed at least 6 months before trial entry. Patients received oral AZD8931 on days 1–4 in a 2-weekly schedule plus FOLFIRI starting on day 1, given every 2 weeks. Patients remained on treatment until disease progression, unless there were intolerable side-effects, treatment delays longer than 3 weeks or withdrawal of consent. If AZD8931 was stopped patients were permitted to continue to receive FOLFIRI alone.

PANTHER adopted a seamless Phase I/II trial design. Phase I design was done using a dose-escalation continual reassessment method (CRM) [30], which was used to evaluate tolerability of FOLFIRI in combination with AZD8931 20 mg bd, 40 mg bd, 80 mg bd and 160 mg bd, using a target probability of toxicity of 33%. A minimum of two patients were required for a dose escalation decision by the Trial Management Group (TMG). The Phase II part of the trial opened as a randomised trial in July 2016 aiming to recruit 40 patients (20 in each arm), which was sufficient to detect, with an 80% power and 1-sided significance level, an assumed 20% difference in the mean percentage change in the tumour size at 12 weeks from baseline between treatment groups with a standard deviation of 30%. Due to projected expiry of the AZD8931 drug supply, the trial was amended in May 2017 to a single-arm single-stage study aiming to recruit a total of 21 patients to evaluate whether the response rate with AZD8931 was improved by 20% from an assumed historical control response rate of 35%, based on an 80% power and a one-sided alpha of 15%. The requirement for biopsy resulted in significant delays in recruitment for this study.

### Outcomes

Dose-limited toxicities including cardiovascular (prolongation of QT interval, congestive cardiac failure or reduction in LVEF) were evaluated from the start of cycle 1 to the end of cycle 2. Disease status was assessed by CT scan and evaluated using RECIST v1.1 at baseline, week 12 and every 12 weeks during treatment. Progression-free survival (PFS) and OS were measured. Adverse events were assessed according to CTCAE v4.03 until 30 days post last trial treatment administration.

Pharmacokinetic and pharmacodynamic studies were carried out as per protocol. Exploratory biological endpoints included analysis of DNA damage and repair by the single-cell gel electrophoresis (Comet) assay in peripheral blood mononuclear cells (PBMCs), profiling of patient serum for ligands including amphiregulin (AR), epidermal growth factor (EGF), heparin-binding EGF (HBEGF) and transforming growth factor alpha (TGF-α) and analysis of circulating exosomes. ERBB dimerisation status was assessed in circulating exosomes.

PBMCs were isolated from patients in Phase I and analysed for the effect of AZD8931 on DNA damage and repair using single-cell gel electrophoresis (Comet) assay [31]. Serum from patients in Phase II were analysed for the presence of AR, EGF, HBEGF and TGF-α biomarkers using the Aushon Ciraplex multiplex ELISA system.

Exosomes from plasma of 13 patients in the Phase I trial, before and after their first dose of AZD8931 were extracted using an in-house optimised ultracentrifugation protocol (Supplementary Methods). Concentration and size distribution of exosome preparations were measured using the Nanosight LM10-HS (Nanosight Ltd, Amesbury, UK), prior to being analysed for proteins of interest including EGFR, HER2, HER3 and S100A9, using dot blot analysis. Exosomal HER2-3 dimers were analysed using FRET/FLIM, and FRET efficiencies was calculated for each patient before and after treatment with AZD8931. FRET score was classified as positive or negative according to whether a significant dimer score could be detected at that patient time point (Supplementary Methods).

### Data analysis

Statistical analysis in this study was mostly descriptive. The complete response/partial response (PR) rate was reported along with 70% CI. The change in tumour size at 12 weeks (or time of progression) and adverse events were reported in terms of mean and percentages, respectively. OS and PFS were reported using Kaplan–Meier curves along with median time to event and 12- and 24-month event-free rates. Treatment comparisons in terms of OS and PFS were reported using hazard ratios, 95% confidence interval (CI) and *p* values (2-sided) derived from Cox regression. Pharmacokinetic data was described using means between AZD8931 dosing with and without FOLFIRI. 95% CIs were used when appropriate. Marker dot blot change was calculated as percentage change compared to the baseline timepoint 1. FRET class was compared between timepoints. Data analysis was done using STATA 15.1 and R 4.0.

## Results

**Fig. 1:**
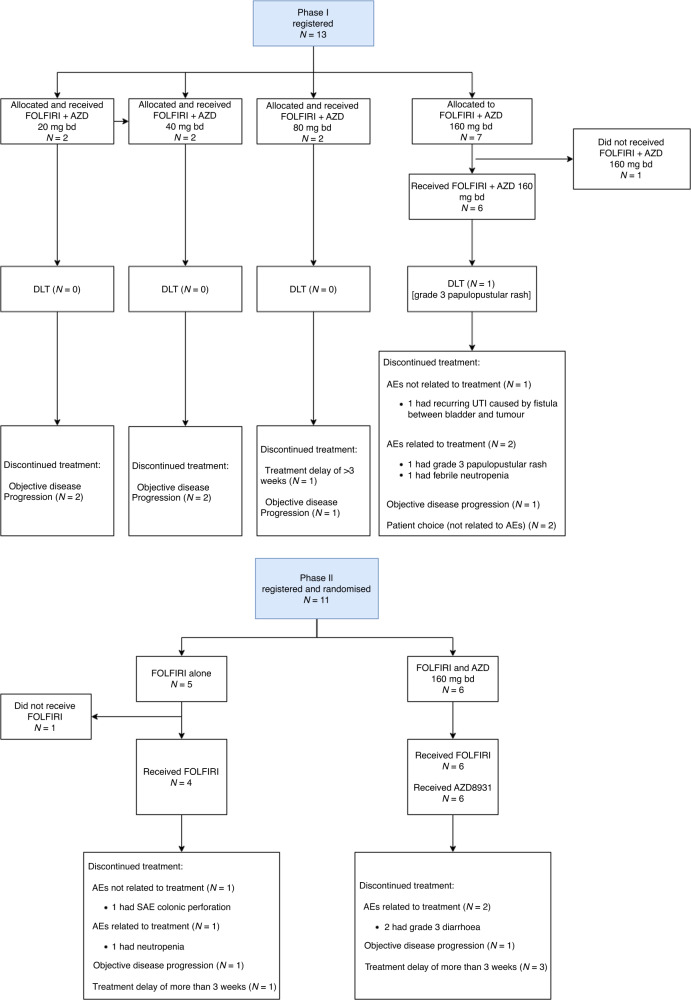
Study flow chart for Phase I and phase II cohorts.

The Phase I part of the trial recruited a total of 13 patients between July 2014 and April 2016 from four hospital sites (Fig. 1). Of those, 12 were evaluable for DLT: 2 patients were treated at each of the 20, 40 and 80 mg bd dose levels and 6 patients were treated at the 160 mg bd dose level. A total of 11 patients were recruited into Phase II from 4 sites between September 2016 and June 2017. All 6 patients registered to FOLFIRI and AZD8931 and 4 of the 5 patients allocated to FOLFIRI alone started treatment (1 patient decided to withdraw before start of FOLFIRI). A total of 18 patients (12 in Phase I and 6 in Phase II) received FOLFIRI and AZD8931 and are the evaluable population in this study. Baseline characteristics are outlined in Table 1. All 18 patients had measurable target lesions.

**Table 1:** Baseline characteristics.

Baseline characteristics	Any AZD dose
	*N* = 18
	*N* (%)
Age in years	
Median (range)	63 (37–77)
BMI in kg/m^2^	
Median (range)	26 (19–32)
Sex	
Male	15 (83%)
Female	3 (17%)
WHO performance status	
0 (fully active)	13 (72%)
1 (restricted in physical activity)	5 (28%)
Previous surgery	
No	5 (28%)
Yes	13 (72%)
Stent in situ	
No	14 (78%)
Yes	3 (17%)
Unknown	1 (6%)
Previous radiotherapy for localised disease	
No	15 (83%)
Yes	3 (17%)
Previous adjuvant chemotherapy	
No	13 (72%)
Yes	5 (28%)
Previous hormonal/biological/immunological therapy	
No	18 (100%)

### Treatment compliance

The reasons for treatment discontinuation were: progressive disease (PD)—7/18 patients, 39%; adverse events related to treatment (AZD8931 and/or FOLFIRI) - 4/18, 22%; (2 diarrhoea, 1 skin reaction, 1 febrile neutropenia); treatment delay of more than 3 weeks due to unrelated reasons - 4/18, 22%, patient choice - 2/18, 11% and adverse events not related to treatment - 1/18, 6%.

### Toxicity and safety

Only 1 patient receiving AZD8931 160 mg bd experienced a grade 3 DLT (papulopustular rash), establishing the AZD8931 160 mg bd as the recommended dose to be tested in the cohort expansion. There were no grade 5 adverse events. A total of 13 (72%) patients had at least a grade 3 adverse event. Of those, 4 (22%) had a grade 4 adverse event: 1 patient received AZD8931 80 mg bd and had sepsis and thromboembolic event; 1 patient had colonic perforation and 2 patients had sepsis in the AZD8931 160 mg bd arm. The most common grade 3 events among patients who received AZD8931 and FOLFIRI were diarrhoea (4, 33%), neutrophil count decreased (4, 33%), hypertension (3, 25%) and GGT increased (3, 25%).  Table 2 presents the reported adverse events related to AZD8931.

**Table 2:** Adverse events reported as related to AZD8931 by grade.

Adverse events reported as related to AZD8931 (worst grade per patient)	Any AZD dose level			
	*N* = 18			
	*N* (%)			
	Grade 1	Grade 2	Grade 3	Grade 4
Blood and lymphatic system disorders				
Anaemia	1 (6%)	2 (11%)	–	–
Febrile neutropenia	–	–	1 (6%)	–
Other blood and lymphatic system disorders	1 (6%)	–	–	–
Eye disorders				
Blurred vision	3 (17%)	–	–	–
Dry eye	2 (11%)	–	–	–
Eye pain	1 (6%)	–	–	–
Other eye disorders	1 (6%)	–	–	–
Gastrointestinal disorders				
Abdominal pain	2 (11%)	–	–	–
Constipation	4 (22%)	–	–	–
Diarrhoea	7 (39%)	–	4 (22%)	–
Dry mouth	1 (6%)	–	–	–
Dyspepsia	1 (6%)	–	–	–
Flatulence	2 (11%)	–	–	–
Gastro-oesophageal reflux disease	2 (11%)	–	–	–
Mucositis oral	3 (17%)	2 (11%)	1 (6%)	–
Nausea	6 (33%)	3 (17%)	–	–
Oral pain	1 (6%)	–	–	–
Other gastrointestinal disorders	1 (6%)	–	–	–
Vomiting	1 (6%)	–	–	–
General disorders and administration site conditions				
Chills	1 (6%)	–	–	–
Fatigue	7 (39%)	8 (44%)	1 (6%)	–
Fever	–	1 (6%)	–	–
Flu-like symptoms	2 (11%)	–	–	–
Infections and infestations				
Lip infection	1 (6%)	–	–	–
Papulopustular rash	3 (17%)	1 (6%)	1 (6%)^a^	–
Paronychia	1 (6%)	–	–	–
Sepsis	–	–	–	1 (6%)
Investigations				
Neutrophil count decreased	–	–	3 (17%)	–
Other Investigations	2 (11%)	–	–	–
Weight loss	–	1 (6%)	–	–
Metabolism and nutrition disorders				
Anorexia	5 (28%)	1 (6%)	–	–
Nervous system disorders				
Concentration impairment	1 (6%)	–	–	–
Dysgeusia	3 (17%)	–	–	–
Headache	1 (6%)	–	–	–
Lethargy	1 (6%)	–	–	–
Movements involuntary	1 (6%)	–	–	–
Renal and urinary disorders				
Cystitis noninfective	1 (6%)	–	–	–
Respiratory, thoracic and mediastinal disorders				
Dyspnoea	–	1 (6%)	–	–
Epistaxis	2 (11%)	–	–	–
Hiccups	1 (6%)	–	–	–
Hoarseness	1 (6%)	–	–	–
Other respiratory, thoracic and mediastinal disorders	1 (6%)	–	–	–
Skin and subcutaneous tissue disorders				
Alopecia	2 (11%)	–	–	–
Dry skin	8 (44%)	–	–	–
Nail loss	1 (6%)	–	–	–
Other skin and subcutaneous tissue disorders	1 (6%)	–	–	–
Pain of skin	1 (6%)	–	–	–
Palmar–plantar erythrodysesthesia syndrome	1 (6%)	–	–	–
Periorbital oedema	1 (6%)	–	–	–
Pruritus	1 (6%)	–	–	–
Rash acneiform	7 (39%)	1 (6%)	–	–
Rash maculo-papular	2 (11%)	–	–	–
Scalp pain	2 (11%)	–	–	–
Vascular disorders				
Thromboembolic event	–	1 (6%)	1 (6%)	1 (6%)
Number of patients experiencing any adverse event				
Any adverse event	4 (22%)	6 (33%)	7 (39%)	1 (6%)

Note: The numbers represent the count (%) of patients experiencing an adverse reaction based on their worst grade. The same patient can be counted across multiple rows because a patient can experience multiple events.

^a^Dose-limiting toxicity.

### Response of patients on study

Table 3 shows the best overall response rate by cohort.

**Table 3:** Best overall response.

Best overall response	FOLFIRI and AZD8931			
	AZD 20 mg bd	AZD 40 mg bd	AZD 80 mg bd	AZD 160 mg bd
	*N* = 2	*N* = 2	*N* = 2	*N* = 12
	*N* (%)	*N* (%)	*N* (%)	*N* (%)
Partial response	1 (50%)	1 (50%)	1 (50%)	3 (25%)
Stable disease	–	1 (50%)	–	8 (67%)
Progressive disease	1 (50%)	–	–	1 (8%)
Not assessed	–	–	1 (50%)^a^	–

^a^Patient died prior to 12-week scan.

The best overall response rate among the 18 patients treated with AZD8931 is 33% (exact 95% CI: 13–59%, 70% CI: 21–48%). The best overall response rate among the 12 patients treated with AZD8931 at 160 mg bd is 25% (exact 95% CI: 5–57%, 70% CI: 11–44%). Based on the estimated rate and on the estimated 70% CI, there is no evidence, at 1-sided 15% significance level, that the response rate among those who received AZD8931 160 mg bd (from Phase I and Phase II) was higher than the assumed response rate of 35% in historical controls.

The mean % change in the sum of longest diameters at 12 weeks from baseline among all 18 patients who received AZD8931 was −10.02% (95% CI: −22.97 to 2.92) and among the 12 patients who received 160 mg bd was −7.60% (95% CI −21.82 to 6.62). One patient who received 160 mg bd had a 21% shrinkage in target lesions at 12 weeks but also had new lesions identified and was therefore assessed as having progressive disease.

### Time-to-event outcomes

**Fig. 2:**
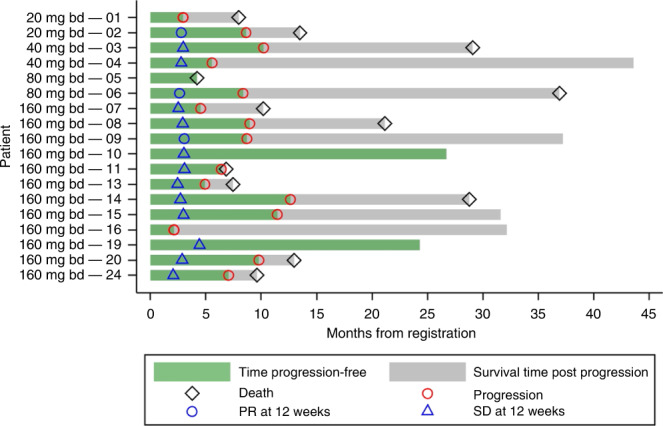
Swimmer plot of 18 AZD8931-treated patients. The swimmer plot depicts individual patient responses at 12 weeks, duration until disease progression, and duration until death or last follow-up.

The median follow-up time among the 18 patients registered who received AZD8931 is 32.2 months. Figure 2 shows key information about time-to-event outcomes.

**Fig. 3:**
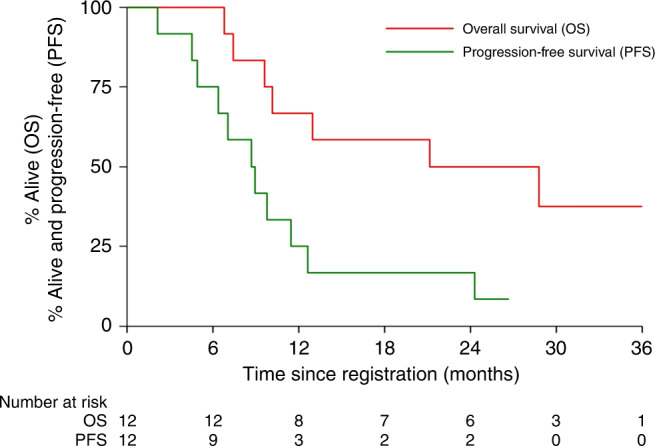
Kaplan-Meier for progression-free survival (PFS) and for overall survival (OS) among patients who received FOLFORI and AZD8931 160 mg bd. PFS events were defined as disease progression or death, whichever occurred first. OS events include deaths from any cause. Number at risk are given under the plot.

A total of 17 (94%) patients had a reported PFS event (progression or death) and 12 (67%) deaths have been reported. The cause of death was disease progression for 6 patients, 2 due to sepsis, 1 pneumonia and 3 due to other unknown reasons. Figure 3 presents the PFS and OS curves among the 12 patients who received 160 mg bd AZD8931.

Among this group, the median PFS time was 8.7 months, and the 12 and 24 months PFS rate was 25% (6–50%) and 17% (3–41%), respectively; the median OS was 21.2 months, and the 12- and 24-month OS rate 67% (34–86%) and 50% (21–74%), respectively. Patients who received any AZD dose presented improved OS when compared to patients who received FOLFIRI alone (Supplementary Fig. 1A, HR: 0.23 (95% CI: 0.06–0.84), *p* = 0.03) but PFS was similar between these two groups (Supplementary Fig. 1B, 0.81 (95% CI: 0.27–2.49), *p* = 0.72).

### Pharmacokinetics

Plasma was isolated from whole blood collected pre-treatment and at 1.5, 2.5, 4, 8, 10 and 24 h post-FOLFIRI infusion, at cycle 1 day and cycle 3 day 1. The mean AUC (0-t) for AZD8931 at 20 mg bd was 11,395.5 and 12,493.78 ng/mL, at 40 mg bd was 20,795.4 and 22,864 ng/mL, at 80 mg bd was 55,156.75 and 72,049.15 ng/mL and at 160 mg bd was 137,221.39 and 185,184.96 ng/mL, when AZD8931 was administered without and with FOLFIRI, respectively. The mean AUC (0–*t*) for irinotecan when administered without and with AZD8931 was not substantially different across dose levels (598.5  vs 511.7 µg/mL at 20 mg bd; 547.8 vs 537.2 µg/mL at 40 mg bd, 765.0 vs 774.7 µg/mL at 80 mg bd; and 716.1 vs 750.0 µg/mL at 160 mg bd). Similar patterns were observed for the other PK parameters. Therefore, combination treatment did not alter pharmacokinetics of either AZD8931 or irinotecan.

### Pharmacodynamic analysis

No significant difference was observed in DNA damage as measured as Olive tail moment (OTM) (µm) between patient dose cohorts of AZD8931 (Supplementary Fig. 2).

Ligand analysis was performed on a total of 22 samples received for 9 patients but due to the small numbers it is not possible to present any results or conclusions to this.

### Exosomal protein and HER 2-3 dimer analysis

Exosomes from plasma of 13 patients before and after their first dose of AZD8931 were extracted and analysed for proteins of interest and HER2-3 dimer quantity. Plasma-derived exosomes were characterised by the presence of exosome surface marker proteins CD63 and ALIX. HER proteins were consistently detected from circulating exosomes and their quantities changed with treatment (Supplementary Fig. 3). Treatment induced changes in FRET dimer scores were also determined and patients were classified as FRET positive or negative according to the raw FRET efficiency values (Table 4 and Supplementary Fig. 4).

**Table 4:** Dimer score changes before and after first dose of AZD8931.

Patient number	HER1:HER3		HER2:HER3		Response at 12 weeks
	Baseline	After AZD8931	Baseline	After AZD8931	
1	Positive	Negative	Negative	Positive	PD
2	Negative	Negative	Positive	Negative	PR
3	–	–	Negative	Negative	SD
4	–	–	Negative	Negative	SD
5	Negative	Negative	Negative	Negative	–
6	Negative	Negative	Positive	Negative	PR
7	–	–	Positive	Positive	SD
8	–	–	Negative	Negative	SD
9	Negative	Negative	Negative	Negative	PR
10	Negative	Negative	–	–	SD
11	Negative	Positive	Negative	Negative	SD
12	–	–	–	–	–
13	Negative	Negative	–	–	SD

We observed changes in HER markers (Table 5) and FRET dimer scores (Table 4) between time points 1 and 3.

**Table 5:** Protein expression changes before and after first dose of AZD8931.

Patient number	S100		HER1		HER2		HER3		Response at 12 weeks
	Baseline	% change after AZD8931	Baseline	% change after AZD8931	Baseline	% change after AZD8931	Baseline	% change after AZD8931	
1	1.10	−9.90	0.96	−31.00	0.92	−24.00	0.92	73.00	PD
2	1.30	12.00	0.51	12.00	1.10	33.00	1.10	13.00	PR
3	0.82	−7.00	0.99	−2.40	1.40	−22.00	1.40	−50.00	SD
4	1.10	−13.00	0.47	−34.00	0.78	−15.00	0.78	−30.00	SD
5	0.79	19.00	1.90	−14.00	1.10	−8.00	1.10	−89.00	–
6	–	–	–	–	–	–	–	–	PR
7	0.66	–	0.39	–	0.77	–	0.77	–	SD
8	0.54	3.60	0.78	−35.00	0.87	−15.00	0.87	−41.00	SD
9	–	–	–	–	–	–	–	–	PR
10	1.40	−6.10	0.69	−14.00	0.87	66.00	0.87	68.00	SD
11	2.10	–	1.00	–	1.20	–	1.20	–	SD
12	–	–	–	–	–	–	–	–	–
13	0.94	–	0.48	–	1.30	–	1.30	–	SD

Two patients of the three who had a PR at 12 weeks had a decreased HER2-3 dimer score between those time points; no other patients showed this response. The one patient who had PD at 12 weeks had an increased HER2-3 dimer score and a decreased EGFR/HER-3; again no other patient had this response. All other patients had stable disease at 12 weeks and experienced no change in any dimer score.

## Discussion

This study showed that the administration of pulse high-dose AZD8931 160 mg bd was well tolerated being associated with a relatively low toxicity profile. However, the best overall response rate among the patients treated with AZD8931 160 mg bd was 25% (all PRs) and therefore it was not shown to be improved when compared to the assumed historical control rate of 35%.

Preclinical studies show that EGFR inhibition enhances responses to DNA-interactive chemotherapies by a variety of mechanisms including inhibition of DNA repair [32]. However, the use of small molecules to inhibit the EGFR pathway has had limited success. A Phase II trial of gefitinib in combination with chemotherapy resulted in responses in a significant proportion of patients but drug-induced toxicities including rash and diarrhoea prevented further development of this strategy [33].

A potentially important factor in the lack of success in these combinations may be the cell cycle effects of TKI treatment of cancers. Gefitinib induces a cell cycle arrest in G1 phase with chronic administration and this may inhibit the effects of agents which act preferentially in S phase such as irinotecan. For example, in a preclinical study in breast cancer of the effects of gefitinib, short-term administration was associated with enhanced DNA damage when combined with DNA-interactive chemotherapies, whereas chronic exposure of cells for 48 h resulted in G1 arrest and diminished efficacy of the combination [34]. The current trial using pulsed high doses of AZD8931 immediately prior and early during cytotoxic chemotherapy was designed to minimise effects on cell cycle distribution of cancer cells.

From the limited and preliminary data in this study, there is no evidence that AZD8931 improves the rate of response when compared with historical control data. However, these preliminary data suggest that giving AZD8931 in combination with FOLFIRI is feasible and tolerable. In the FOCUS4-D study of metastatic colorectal cancer, patients with tumours that were stable or responding to cytotoxic chemotherapy received single agent AZD8931 40 mg bd continuously, and there was no PFS benefit of AZD8931 compared with placebo [8]. Despite using doses that were between 4 and 8 times higher than those previously used, the toxicity attributed to AZD8931 in this study was lower than that reported in studies using more prolonged dosing schedules and was in general well-tolerated [8, 35]. Thus, high-dose pulse scheduling in combination with standard doses of cytotoxic chemotherapy appears feasible.

Although there have been many studies investigating inhibition of EGFR in CRC little is known regarding the dynamics of the EGFR pathway in human cancers. We have previously demonstrated that the combined use of HER2-HER3 dimer imaging (applied to FFPE pathological specimens) and conventional mutation analyses can identify in a predictive manner the small subclass of metastatic CRC patients (15%) who will have better prognosis following chemotherapy/cetuximab treatment [29]. One hypothesis is that low affinity EGFR ligands amphiregulin (AREG) and epiregulin (EREG) which are positively predictive of anti-EGFR treatment outcome, may induce prolonged and more widespread signalling, involving the formation of non-EGFR heterodimers such as HER2-3 [36, 37]. In colorectal cell lines, we observed that treatment with AZD8931 resulted in increased HER2-3 dimer formation in cetuximab-sensitive cell line LIM1215 and reduced HER2-3 dimer formation in DLD1 (KRAS WT) (Supplementary Fig. 5). Previous work in our group also demonstrated, for the first time, increased HER2-3 dimerisation upon cetuximab treatment in colon cancer cells [28].

In this study, we observed both increase and decrease of HER 2-3 dimer formation in patients after treatment with AZD8931, correlating to findings in cell line data. Our group have previously reported in breast cancer HER2-3 dimer quantification in formalin-fixed paraffin-embedded (FFPE) tumours predicted metastatic relapse post-surgery that was independent of HER2 expression [24]. The significance of these findings will need to be explored in future colorectal cancer trials using different HER2 targeted agents.

In the literature, there is a presumption that pan-HER tyrosine kinase inhibitors reduce the formation of homologous/heterodimer of HER receptors [38]. Our results indicate HER2-3 heterodimers measured in blood exosomes can increase or decrease following treatment and our preliminary results indicate that the relationship between this dynamic response and tumour response as assessed by RECIST should be further defined in patients with metastatic colorectal cancer treated with anti-HER therapies. It seems likely that inhibition of multiple members of the HER family will have additional biological effects as compared with EGFR alone. The current study included only a limited number of patients and hence no definitive conclusions can be made regarding the utility of this combination approach. Future studies incorporating combinations of chemotherapy and targeted agents should include measurement of translational endpoints which assess pathway activation.

## Conclusion

This study established that a 4-day pulsed high dose schedule AZD8931 of 160 mg bd is safe and sufficiently tolerated when combined with FOLFIRI. Further studies of TKI sequencing may establish a role for pulsed use of such agents rather than continuous exposure.

## Supplementary information

Supplementary Material

Reproducibility checklist

CONSORT checklist

## Data Availability

Data will be available for sharing after the main trial publication is released. All requests for data will be considered by the Chief Investigator/TMG and, if approved, shared via a data sharing agreement. Decision making factors will include: Data use is in keeping with patient consent. Confidentiality is maintained at all times i.e. no patient identifiable data, such as date of birth will be provided. Data are appropriate for intended purpose. Compliance with legal and ethical requirements is maintained.

## Code availability

The code used for data cleaning and analysis is available upon request from ctc.panther@ucl.ac.uk.
