# Supplementary material for: PANTHER: AZD8931, inhibitor of EGFR, ERBB2 and ERBB3 signalling, combined with FOLFIRI: a Phase I/II study to determine the importance of schedule and activity in colorectal cancer
Source: Br J Cancer. 2022 Nov 9;128(2):245–54. doi: 10.1038/s41416-022-02015-x (PMC9902557; doi:10.1038/s41416-022-02015-x)

**SUPPLEMENTARY MATERIALS**

**Supplementary Methods**

**Serum Exosome Isolation**

Patients’ frozen serum (-80 ºC) was used for exosome isolation using an optimized ultracentrifugation method (Monypenny et al., 2018). Serum samples were thawed on ice, and 500 μl were diluted 1:3 with sterile, 0.45 μm-filtered PBS to make it up to 1.5 ml.

The diluted serum was centrifuged at 300×g for 10 min to remove cell debris, at 5,000×g for 20 min to remove large vesicles and membrane fragments, then at 12,200×g for 30 min to deplete microvesicles (MV), the last step was repeated for an extra 30 min, to prevent any further MV contamination of the exosomal fraction. This was followed by 100,000×g ultracentrifugation for 120 min to pellet exosomes using micro-ultracentrifuge tubes (1.5 ml, Cat No. 357448, Beckman Coulter) in a Beckman Optima-Max XP ultracentrifuge equipped with a TLA-55 rotor (Beckman Coulter). After centrifugation, the supernatant was carefully removed and the pellets washed with sterile PBS. A second 100,000×g ultracentrifugation for 60 min was used and the resulting pellets were resuspended in 100 μl of sterile, 0.45 μm-filtered PBS. All centrifugation steps were performed at 4 °C. From purified exosomal fractions, 5-20 μl were diluted up to 1 ml in PBS and used for nanoparticle tracking analysis (NTA) using a Nanosight LM-14 system (Malvern Ltd) as described previously using constant flow injection. The NTA analysis software was used to calculate the particle population size distribution including the derivation of the population’s modal particle diameter.

## **Peripheral Blood Mononuclear Cell Isolation**

## Peripheral blood mononuclear cells (PBMCs) were isolated from whole blood, taken from patients at the following time points:-

- Pre-treatment: within 7 days of first dose of AZD8931
- Cycle 1 Day 1: before morning dose of AZD8931 + FOLFIRI administration
- Cycle 1 Day 2: before morning dose of AZD8931
- Cycle 3 Day 2: before morning dose of AZD8931

All samples were processed at the clinical trial site. At each time point, 8mL whole blood was withdrawn from the patient and collected into 2 x4mL Vacutainer® CPT tubes (sodium citrate) and mixed gently. Within 30 minutes (min) of blood draw, samples were centrifuged at 1500g for 20 min at room temperature. The fluffy mononuclear layer at the interface of the two layers was removed using a Pasteur pipette and transferred to a 15 mL Falcon tube. 10 mL cold RPMI 1640 media was added and the tube gently inverted and centrifuged immediately at 200g for 5 min at 4°C. The supernatant was discarded and the cell pellet resuspended in 2 mL freezing mixture (foetal calf serum (FCS) containing 10% dimethylsulphoxide (DMSO)) maintained at 4°C. 1 mL cell suspension was then aliquoted into two labelled cryovials and frozen at -70°C or below.

All samples were analysed for DNA strand breaks using the single cell gel electrophoresis (Comet) assay (Hartley, Spanswick, & Hartley, 2011). All procedures were carried out on ice and in subdued lighting. Samples were thawed on ice, diluted to 2.5 x10^4^/ml. After embedding cells in 1% agarose on a precoated microscope slide, the cells were lysed for 1hr in lysis buffer (100 mM disodium EDTA, 2.5 M NaCl, 10 mM Tris-HCl pH 10.5) containing 1% Triton X-100 added immediately before analysis, and then washed every 15 min in distilled water for 1hr. Slides were then incubated in alkali buffer (50mM NaOH, 1mM disodium EDTA, pH12.5) for 45 min followed by electrophoresis in the same buffer for 25min at 18 V (0.6V/cm), 250mA. The slides were finally rinsed in neutralising buffer (0.5 M Tris-HCl, pH 7.5) then phosphate buffered saline. After drying the slides were stained with propidium iodide (2.5 μg/mL) for 30 min then rinsed in distilled water. The slides were then dried and stored in a lightproof box until analysis.

## **Data Analysis and Interpretation: Single cell gel electrophoresis (Comet) Assay**

Images were visualised using an Olympus BX51 inverted microscope with a super high pressure Olympus U-RFL-T mercury lamp using a 580 nm dichroic mirror, 535 nm excitation filter and 645 nm emission filter for propidium iodide staining. The images were captured using a Sony XCD-X710 digital camera and analysed using Komet Analysis software version 5.5 (Andor Technology, UK). For each duplicate slide 25 cells were analysed. The tail moment for each image was calculated using the Komet Analysis software as the product of the percentage DNA in the comet tail and the distance between the means of the head and tail distributions, based on the definition of Olive et al (1990) (Olive, Banath, & Durand, 1990). The greater the Olive tail moment, the greater the level of DNA Damage.

**REFERENCES**

Barber, P. R., Tullis, I. D. C., Pierce, G. P., Newman, R. G., Prentice, J., Rowley, M. I., Vojnovic, B. (2013). The gray institute “open” high-content, fluorescence lifetime microscopes. *Journal of Microscopy*, *251*(2), 154–167. https://doi.org/10.1111/jmi.12057

Barber, Paul R., Ameer-Beg, S. M., Gilbey, J. D., Edens, R. J., Ezike, I., & Vojnovic, B. (2005). Global and pixel kinetic data analysis for FRET detection by multi-photon time-domain FLIM. In *Multiphoton Microscopy in the Biomedical Sciences V* (Vol. 5700, p. 171). SPIE. https://doi.org/10.1117/12.590510

Hartley, J. M., Spanswick, V. J., & Hartley, J. A. (2011). Measurement of DNA Damage in Individual Cells Using the Single Cell Gel Electrophoresis (Comet) Assay. In *Methods in molecular biology (Clifton, N.J.)* (Vol. 731, pp. 309–320). Methods Mol Biol. https://doi.org/10.1007/978-1-61779-080-5_25

Monypenny, J., Milewicz, H., Flores-Borja, F., Weitsman, G., Cheung, A., Chowdhury, R., … Ng, T. (2018). ALIX Regulates Tumor-Mediated Immunosuppression by Controlling EGFR Activity and PD-L1 Presentation. *Cell Reports*, *24*(3), 630–641. https://doi.org/10.1016/j.celrep.2018.06.066

Olive, P. L., Banath, J. P., & Durand, R. E. (1990). Heterogeneity in radiation-induced DNA damage and repair in tumor and normal cells measured using the “comet” assay. *Radiation Research*, *122*(1), 86–94. https://doi.org/10.2307/3577587

**SUPPLEMENTARY FIGURES**

**Supplementary Figure 1A: Overall survival amongst patients who received AZD (phase I and II) versus patients who received FOLFIRI alone (phase II) -** HR: 0.23 (95%CI: 0.06 to 0.84), p=0.03

**Supplementary Figure 1B: Progression-free survival amongst patients who received AZD (phase I and II) versus patients who received FOLFIRI alone (phase II) - 0.81 (95%CI: 0.27 to 2.49), p=0.72**

**Supplementary Figure 2:** Effect of AZD8931 on single strand break DNA damage in PBMCs. The mean olive tail moment (OTM) was calculated for each patient and time point (where available) and grouped per AZD8931 dose (bolus dose, bd day 1-4) cohort: - 20, 40, 80 and 160mg. The greater the OTM, the greater the level of DNA Damage. No significant difference was observed in DNA damage as measured as OTM (µm) between patient dose cohorts of AZD8931 (Figure 1). The level of DNA damage observed across timepoints is considered to be comparable to assay background and no DNA damage was observed in the form of single strand break damage. No inhibition of repair was observed due to lack of initial DNA damage.

**Supplementary Figure 3: Effect of AZD8931 on exosomal proteins.** 200-500µl of plasma were used to extract exosomes at 4 different time points per patient (T1-13), before and after first dose of AZD8931; and 5-7 days later, before and after first cycle of chemotherapy combined with AZD8931. Exosome pellets were resuspended in 20-50µl of PBS. Exosome numbers and quality were examined using Nanosight analysis. 5µl/dot were used for protein analysis. There was insufficient plasma for dot blotting at T11.3 for all protein markers, and T13.3 for ALIX. There were no samples received for T12.3 or T12.4.


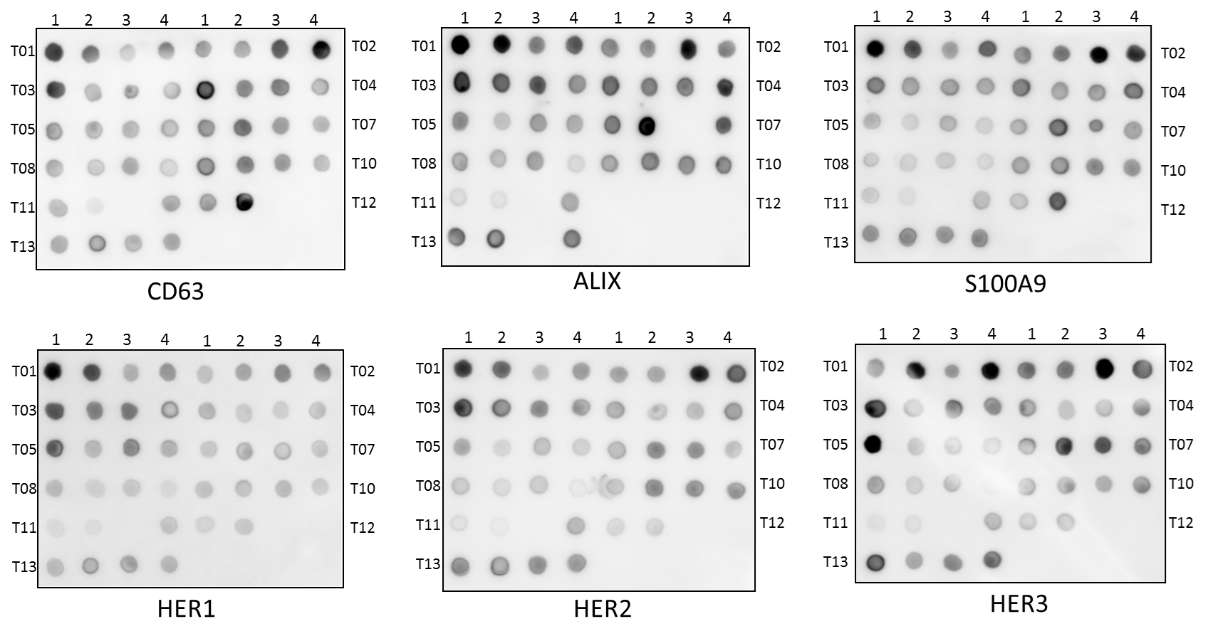


**Supplementary Figure 4: Raw HER13 and HER23 FRET dimer scores for all patients before and after first dose of AZD8931.** Exosomes were attached to glass slides, blocked with 2 % BSA solution in PBS and stained with anti-EGFR-IgG-Alexa Fluor546 (Cetuximab) and anti-ErbB3-IgG-Cy5 (clone REA508, Miltenyi Ltd.) ON at 4 °C. The excess of antibodies was removed by washing with PBS, stained exosomes were the covered with thio-diethanol as a mounting media. The degree of dimerization was quantified with FLIM/FRET. Custom time-domain fluorescence lifetime ‘Open’ microscopes ‘Hooke’ and ‘Galileo’ were used to image exosome slides with a Nikon 20x Plan Fluor objective lens (0.5 NA) as previously described (P. R. Barber et al., 2013). FLIM images of the donor channel were obtained through a filter set optimized for use with Alexa546 (Semrock filters FF01-540/15-25, NT48-492 30R/70T, FF01-593/40-25). Widefield images of the donor channel were acquired through a ‘Cy3’ cube (Ex: 510-560 nm, Em: 573-648 nm) and of the acceptor channel through a ‘Cy5’ cube (Ex: 590-650 nm, Em: 663-738 nm). Slides were imaged with a laser power and scan speed to gain sufficient photon counts within 5 minutes of imaging (typically >100 photon counts per pixel peak). Image dimensions were 256x256 pixels (328x328 microns) by 256 time-resolved bins (spanning 15.0 ns). Optical resolution is approximately 1 micron. Individual exosomes are not resolved but appear as diffraction limited spots. Typically, several thousand exosomes are imaged per field of view, occupying on the order of 10 % of the image pixels. FLIM lifetime measurements were performed with the TRI2 software (version 2.7.8.9, Gray Institute, Oxford) as described previously (Paul R. Barber et al., 2005). Briefly, exosomes were isolated from the background using a threshold that was set by measuring the mode and standard deviation (SD) of the background (based on the modal image intensity and left-hand tail or estimated from Poisson statistics for mode <10 photon counts). The threshold was then set at (mode + 6xSD). This excluded all background-only pixels and aims to ensure sufficient exosome signal in the remainder (since exosome size << 1 pixel, the sample-derived background signal is not displaced by exosome presence, see supplementary exosome intensity modelling). The pixels above the threshold in one field of view were binned into a single transient signal. This transient was fitted with a tri-exponential model of the form: $I=z+A_{1}e^{-t/{\tau_{1}}}+A_{2}e^{-t/{\tau_{2}}}+A_{3}e^{-t/{\tau_{3}}}$. Where I represents signal intensity, z represents a constant background, A represents signal component amplitudes, t represents time and tau the fitted fluorescence characteristic lifetimes. Two lifetimes, τ^­^_2_ and τ^­^_3_, were held constant throughout the analysis at 0.3 ns and 1.0 ns (determined empirically from background test samples) to capture autofluorescence and other interfering components, as previously described, away from the target Alexa546 lifetime of circa 2.5 ns. These components are held constant, and not allowed to vary, to improve fit stability. The value of the free lifetime, τ^­^_1_, was used to represent the lifetime of Alexa546 in the field of view. Between 3 and 5, D and DA sample regions were imaged per patient. Lifetimes for D and DA were averaged (giving τ^­^_D_ and τ^­^_DA_) from the region lifetimes, and a FRET value for the patient was calculated by: 𝐹𝑅𝐸𝑇=1−$\frac{\tau_{\mathrm{DA}}}{\tau_{D}}$.

FRET score was classified as positive or negative according to whether a significant dimer score could be detected for that patient time point, as follows. Five FLIM images with the FRET pair along with five control images were acquired per time point. Images were quality controlled to remove large particles and clumps (> around 500 um) by manual masking. The FRET standard error in the mean (SEM) was calculated from the FRET and control samples, and FRET was classed as positive if the mean value was positive and the SEM did not cross below the zero line.


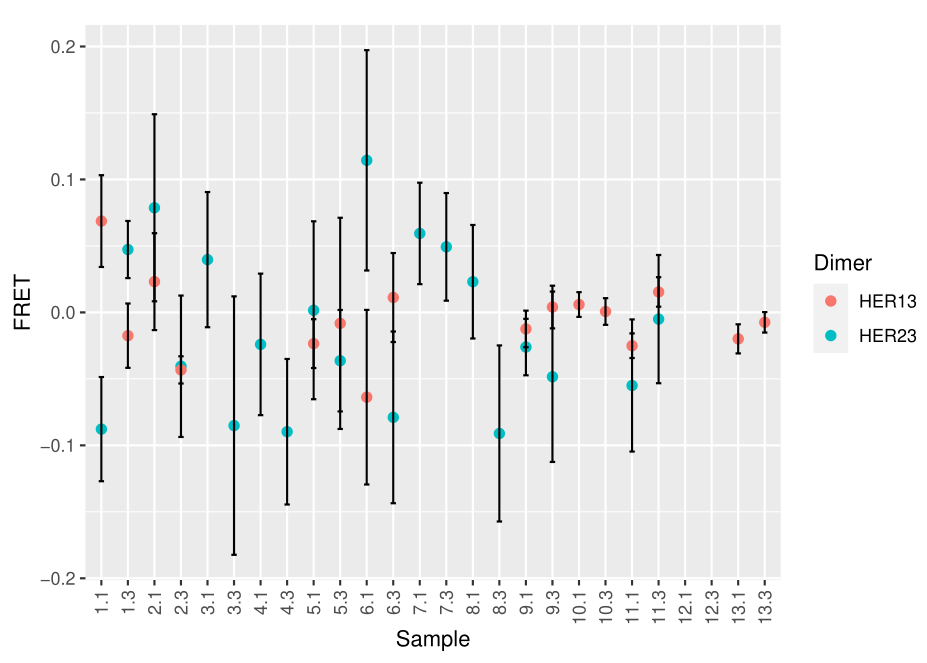


**Supplementary Figure 5: Changes in HER2-3 dimerisation in colorectal cells upon treatment with AZD8931.**

A) LIM1215 cells were plated on coverslips and treated with either cetuximab (15µg/ml or 100nM) or AZD8931 (10µM) for 1 hour, prior to fixation with 4% PFA, and staining with IgG anti-HER2-Cy5 (acceptor) and IgG anti-HER3-Alexa546 (donor). D: Donor alone; DA: Donor with acceptor. Averaged FRET values were calculated on a pixel-by-pixel basis, and the distribution of pixelwise FRET efficiencies plotted for each condition tested. B) Increased HER2-3 dimerisation in LIM1215 with cetuximab and AZD8931. Using paired T-test, p=*0*.043 for cetuximab vs control, *p*=0.003 for AZD8931 vs control. C) Reduced HER2:HER3 dimerisation in DLD1 (KRAS WT) upon AZD8931. *p*=0.077 for cetuximab vs control, *p*=0.026 for AZD8931 vs control. Results are representative of 5 technical repeats each from 2 independent experiments.


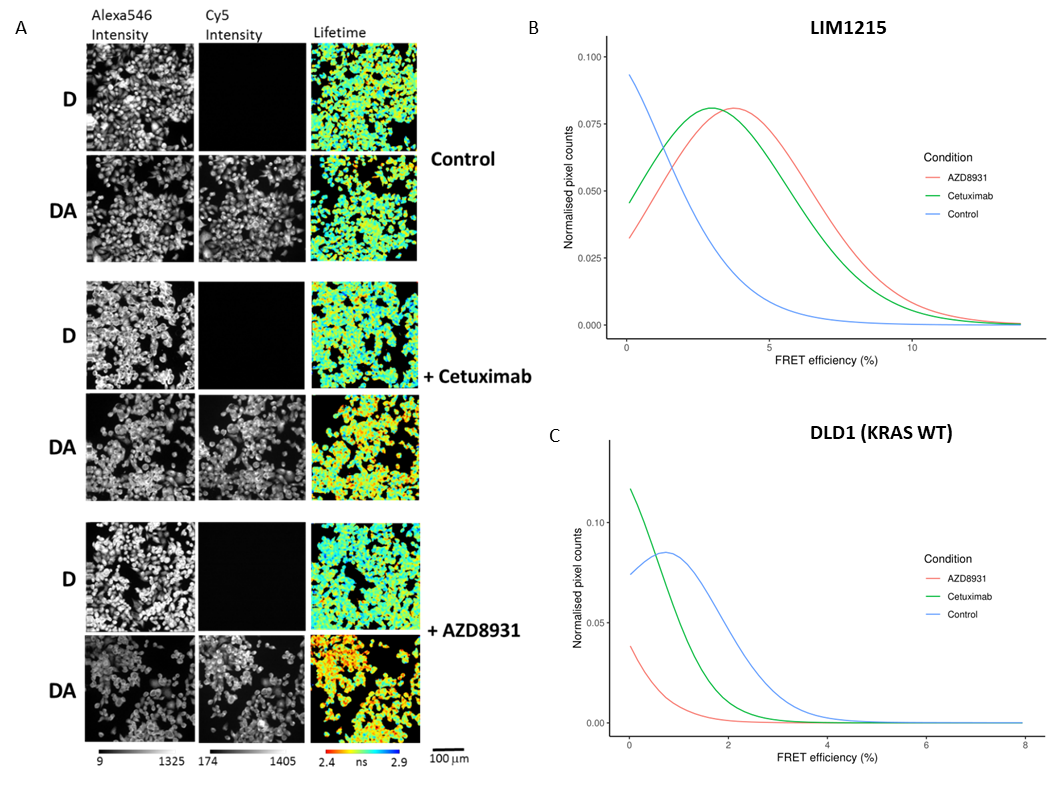

Supplement: Supplementary file 1 — Supplementary Material [file 41416_2022_2015_MOESM1_ESM.docx]
