# Supplementary material for: PANTHER: AZD8931, inhibitor of EGFR, ERBB2 and ERBB3 signalling, combined with FOLFIRI: a Phase I/II study to determine the importance of schedule and activity in colorectal cancer
Source: Br J Cancer. 2022 Nov 9;128(2):245–54. doi: 10.1038/s41416-022-02015-x (PMC9902557; doi:10.1038/s41416-022-02015-x)
Supplement: Supplementary file 2 — Reproducibility checklist [file 41416_2022_2015_MOESM2_ESM.pdf]

Corresponding Author Name: Prof Daniel Hochhauer
Manuscript Number: BJC-A3340844R-A

Reporting Summary

Springer Nature wishes to improve the reproducibility of the work that we publish. This checklist is used to ensure good
reporting standards and to improve the reproducibility. Please respond completely to all questions relevant to your
manuscript. For more information, please read the journal's Guide to Authors.

✓☐ **Check here to confirm that the following information is available in the Material & Methods section:**

- **The exact sample size (*n*)** for each experimental group/condition, given as a number, not a range
- **A description of the sample collection** allowing the reader to understand whether the samples represent technical or biological replicates (including how many animals, litters, culture, etc.)
- **A statement of how many times the experiment shown was replicated in the laboratory**
- **Definitions of statistical methods and measures**: For small sample sizes (n<5) descriptive statistics are not appropriate, instead plot individual data points

  ○ Very common tests, such as *t*-test, simple χ_2 tests, Wilcoxon and Mann-Whitney tests, can be unambiguously identified by name only, but more complex techniques should be described in the methods section

  ○ Are tests one-sided or two-sided?

  ○ Are there adjustments for multiple comparisons?

  ○ **Statistical test results**, e.g., ***P* values**

  ○ Definition of **'center values'** as **median or mean**;

  ○ Definition of **error bars as s.d. or s.e.m. or c.i.**

Please ensure that the answers to the following questions are reported in the manuscript itself. We encourage you to include a specific subsection in the methods section for statistics, reagents and animal models. Below, provide the page number or section and paragraph number.

Statistics and general methods	**Reported in section/paragraph or page #**
1. How was the sample size chosen to ensure adequate power to detect a pre-specified effect size? (Give section/paragraph or page #)	Methods - Participants and study design<br>Page 5, lines 135-147
For animal studies, include a statement about sample size estimate even if no statistical methods were used.	N/A
2. Describe inclusion/exclusion criteria if samples or animals were excluded from the analysis. Were the criteria pre-established? (Give section/paragraph or page #)	N/A
3. If a method of randomization was used to determine how samples/animals were allocated to experimental groups and processed, describe it. (Give section/paragraph or page #)	N/A
For animal studies, include a statement about randomization even if no randomization was used.	N/A
4. If the investigator was blinded to the group allocation during the experiment and/or when assessing the outcome, state the extent of blinding. (Give section/paragraph or page #)	N/A
For animal studies, include a statement about blinding even if no blinding was done.	N/A
5. For every figure, are statistical tests justified as appropriate?	N/A, the main analysis is descriptive;<br>treatment comparisons in terms of OS and PFS were reported using hazard ratios, 95%CI and p-values (2-

	sided) derived from cox regression (Methods – Data Analysis, page 6, lines 178-184)
Do the data meet the assumptions of the tests (e.g., normal distribution)?	N/A, the analysis is descriptive; plots are presented.
Is there an estimate of variation within each group of data?	N/A, results are reported using confidence intervals (Methods – Data Analysis, page 6, lines 178-184)
Is the variance similar between the groups that are being statistically compared? (Give section/paragraph or page #)	This is a single arm study.<br>(Results – Time to event outcome, page 8, lines 244-247 presents a secondary analysis where AZD patients are compared statistically to FOLFORI alone patients in terms of OS and PFS)
Reagents	**Reported in section/paragraph or page #**
6. Report the source of antibodies (vendor and catalog number)	N/A
7. Identify the source of cell lines and report if they were recently authenticated (e.g., by STR profiling) and tested for mycoplasma contamination	N/A
Animal Models	**Reported in section/paragraph or page #**
8. Report species, strain, sex and age of animals	N/A
9. For experiments involving live vertebrates, include a statement of compliance with ethical regulations and identify the committee(s) approving the experiments.	N/A
Human subjects	**Reported in section/paragraph or page #**
11. Identify the committee(s) approving the study protocol.	Additional information - Ethics approval and consent to participate<br>Page 12
12. Include a statement confirming that informed consent was obtained from all subjects.	Additional information - Ethics approval and consent to participate<br>Page 12
13. For publication of patient photos, include a statement confirming that consent to publish was obtained.	N/A
14. Report the clinical trial registration number (at ClinicalTrials.gov or equivalent).	Abstract<br>Page 2
Data deposition	**Reported in section/paragraph or page #**
17. Provide accession codes for deposited data. Data deposition in a public repository is mandatory for:<br>a. Protein, DNA and RNA sequences<br>b. Macromolecular structures<br>c. Crystallographic data for small molecules<br>d. Microarray data	N/A
18. If computer code was used to generate results that are central to the paper's conclusions, include a statement in the Methods section under "**Code availability**" to indicate whether and how the code can be accessed. Include version information as necessary and any restrictions on availability.	Methods - Code availability<br>Page 7, Lines 189-190
